# Munsell Soil Colour Prediction from the Soil and Soil Colour Book Using Patching Method and Deep Learning Techniques

**DOI:** 10.3390/s25010287

**Published:** 2025-01-06

**Authors:** Sadia Sabrin Nodi, Manoranjan Paul, Nathan Robinson, Liang Wang, Sabih ur Rehman, Muhammad Ashad Kabir

**Affiliations:** 1 School of Computing, Mathematics and Engineering, Charles Sturt University, Bathurst, NSW 2795, Australia; mpaul@csu.edu.au (M.P.); sarehman@csu.edu.au (S.u.R.); akabir@csu.edu.au (M.A.K.); 2 Cooperative Research Centre for High Performance Soils, Callaghan, NSW 2308, Australia; n.robinson@federation.edu.au (N.R.); liang.wang@newcastle.edu.au (L.W.); 3 Centre for eResearch and Digital Innovation, Federation University, Mount Helen, VIC 3350, Australia; 4 Global Centre for Environmental Remediation, The University of Newcastle, Callaghan, NSW 2308, Australia

**Keywords:** agriculture, computer vision, mobile phone, augmentation

## Abstract

Soil colour is a key indicator of soil health and the associated properties. In agriculture, soil colour provides farmers and advises with a visual guide to interpret soil functions and performance. Munsell colour charts have been used to determine soil colour for many years, but the process is fallible, as it depends on the user’s perception. As smartphones are widely used and come with high-quality cameras, a popular one was used for capturing images for this study. This study aims to predict Munsell soil colour (MSC) from the Munsell soil colour book (MSCB) by using deep learning techniques on mobile-captured images. MSCB contains 14 pages and 443 colour chips. So, the number of classes for chip-by-chip prediction is very high, and the captured images are inadequate to train and validate using deep learning methods; thus, a patch-based mechanism was proposed to enrich the dataset. So, the course of action is to find the prediction accuracy of MSC for both page level and chip level by evaluating multiple deep learning methods combined with a patch-based mechanism. The analysis also provides knowledge about the best deep learning technique for MSC prediction. Without patching, the accuracy for chip-level prediction is below 40%, the page-level prediction is below 65%, and the accuracy with patching is around 95% for both, which is significant. Lastly, this study provides insights into the application of the proposed techniques and analysis within real-world soil and provides results with higher accuracy with a limited number of soil samples, indicating the proposed method’s potential scalability and effectiveness with larger datasets.

## 1. Introduction

Soil colour is one of the most used indicators of soil health and its properties. It can be useful for identifying soil organic matter, mineral composition, and physical and chemical properties [1]. Classification of soil colour is often performed using a Munsell Soil Colour Book (MSCB), and has been used extensively in US soil since 1949 [2]. The Munsell colour system is based on three components, which are Hue (shade), Value (lightness), and Chroma (saturation). Soil surveyors, scientists and farmers typically use the MSCB in their field research and the morphological properties [3]. The Australian Soil and Land Survey Field Handbook explains the detailed procedure of using MSCB [4].

Traditionally, scientists determine soil colour by matching a soil with the MSCB; therefore, the exercise entirely depends on human visual liking, and it varies from person to person [5]. According to [6], there is a similarity between human eyes and the spectra of seven MSCB colour chips. Determining soil colour can never be assured, as it depends on human judgement and various external lighting conditions and time, as highlighted by [7,8,9,10,11]. Even studies such as [12,13] were performed to estimate soil content and carbon from satellite imagery by using multiple learning based models. Previous studies for soil colour determination were performed under experimental environments [14] and with the help of external device sensors [15]. As an example, ref. [16] used an external attachment kit with a smartphone which includes external lenses, and calibration cards for soil colour determination. Similarly, the authors of [17,18] designed a web application by employing a professional camera for determining Munsell soil colour, which is costly and not ideal for variable field conditions.

Digital cameras have been employed in various studies for estimating soil organic matter from captured images [19,20]. Numerous smartphones are currently available with top-graded cameras that are similar to professional digital cameras. Recent research, like [21,22], shows the use of smartphones in the health and ophthalmology domain is becoming popular. Smartphones are the most used technology in the world, and almost everybody owns at least one of them. People rely on mobile phone applications for many purposes. A group of researchers [23] analysed existing soil-related applications published both in iOS and Android platforms, and proved that smartphone applications have become a rising use for soil classification. Authors of [24] performed a multi-algorithm comparison for predicting soil organic carbon and moisture content using a smartphone. Research in [14] found that smartphones were reliable and cost-effective in the prediction of soil colour, performing better than human judgement.

In [25], the authors confer on the influence of weather conditions on the field for visually determining MSC. Likewise, observing MSC depends on the user’s experience and vision. Therefore, studies are emerging recently to measure MSC accurately without human errors and unavoidable weather errors. Smartphones come equipped with a built-in GPS, high-quality cameras, sensors and more. Recent studies show a huge potential for mobile phone usage in various fields like agriculture, health, and food. The researchers in [26] proved the importance of smartphone cameras in the field of agricultural industry. One study [27] showed a remarkable accuracy for MSC prediction by introducing a colour intensity relationship between images captured by Nix Pro and smartphones. Here, two different smartphones were deployed to evaluate that the colour intensity relationship and its variability on different smartphones. A wide range of research has already been performed using smartphone cameras in different domains like agriculture, soil and plant health. Studies like [16,28] were performed to classify soil type and soil water erosion. Some studies are performed for the betterment of agriculture and better harvest by determining crop seed [29], detecting microbial contamination, and identifying weeds [30,31,32]. Study [33] showed the huge potential of smartphone cameras for determining soil health and fertility. Another study [34] was performed for several soil variables including carbon, pH, nitrogen, etc. Here, estimations for the experiment were undertaken using constant lighting conditions and by maintaining constant distance and angle between the camera and soil sample. Therefore, it is obvious that many factors, including colour, weather, and lighting conditions, need to be considered when estimating soil properties.

Deep learning is a form of machine learning that uses a multi-layered neural network that can be used for complex decision-making and prediction. It can learn from multi-level unstructured data and extract information and features [35]. It has been used widely for image recognition [36]. In recent years, the usage of deep learning has been observed in multiple studies in the soil domain. Another study by [37] has used Convolutional Neural Network (CNN)-type deep learning for soil classification. Studies [38,39] investigated a range of machine learning and deep learning techniques for predicting soil nutrients, including soil moisture, soil organic carbon, and nitrogen content, using data from hyperspectral visual band. Some implementations of deep learning systems can also be seen in soil domains, such as accurate prediction of soil texture (image-based) [40], soil properties [41], and soil moisture [42]. All these studies proved deep learning techniques achieve better accuracy and prediction for soil properties. Therefore, it is understandable that image-based deep learning is an ideal candidate for soil colour prediction.

To implement deep learning models, significant quantities of data are required to enable algorithms in machine learning to identify the patterns of the images of soil colour and predict them. Data augmentation is a powerful way to increase sample size without overfitting the model. Study [43] provides a broader knowledge of how data augmentation can increase the performance of deep learning models by expanding a limited dataset. For this study to make the dataset suitable for deep learning models, a patching method was proposed. By using a patching method, the sample size increased, and the prediction accuracy increased at a higher rate. Previous studies, including [44,45,46], have shown promising results using data augmentation for implementing deep learning techniques. The need to use the patching method has been elaborated in Section 2.2. To demonstrate the practical application of the proposed method of this study in real-world soil scenarios, the approach was implemented on a small number of soil samples. The key contributions of this work are as follows:

- Finding the most suitable deep learning model for MSC prediction by deploying several state-of-the-art methods;
- Analysing patching method for data preparation;
- Comparing and analysing page-level and chip-level soil colour prediction accuracy;
- Demonstrating the patching method in real soil condition.

## 2. Methodology

**Figure 1:**
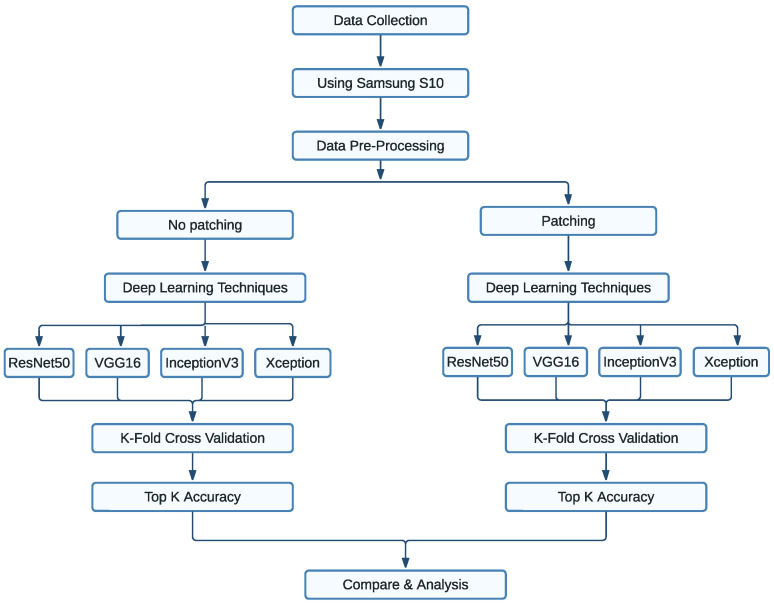
Flow diagram of the proposed process.

Figure 1 shows the methodological process followed for this study. To predict MSC, first, data were collected using a smartphone and pre-processed images before using them for deep learning models, and were named as the primary dataset. The primary and patched datasets were analysed in two categories: (1) Page/Hue-level prediction, and (2) chip-level prediction. The analysis has also determined which deep learning technique is working best for the primary dataset. For analysis, the test accuracy and Top 5 test accuracy were calculated for both patched and primary data using all four deep learning techniques and 10-fold cross-validation. Test accuracy gives the percentage of positive prediction, and the Top 5 accuracy provides information about finding the exact match on the first 5 predictions. As some colour chips are almost identical to each other, agreement with any of the Top 5 predictions will also be accepted. The standard deviation, precision, recall and f1 score were also analysed to determine the best method for MSC prediction. These attributes compare the prediction with test references and ensure which method performs better.

### 2.1. Data Collection and Pre-Processing

**Figure 2:**
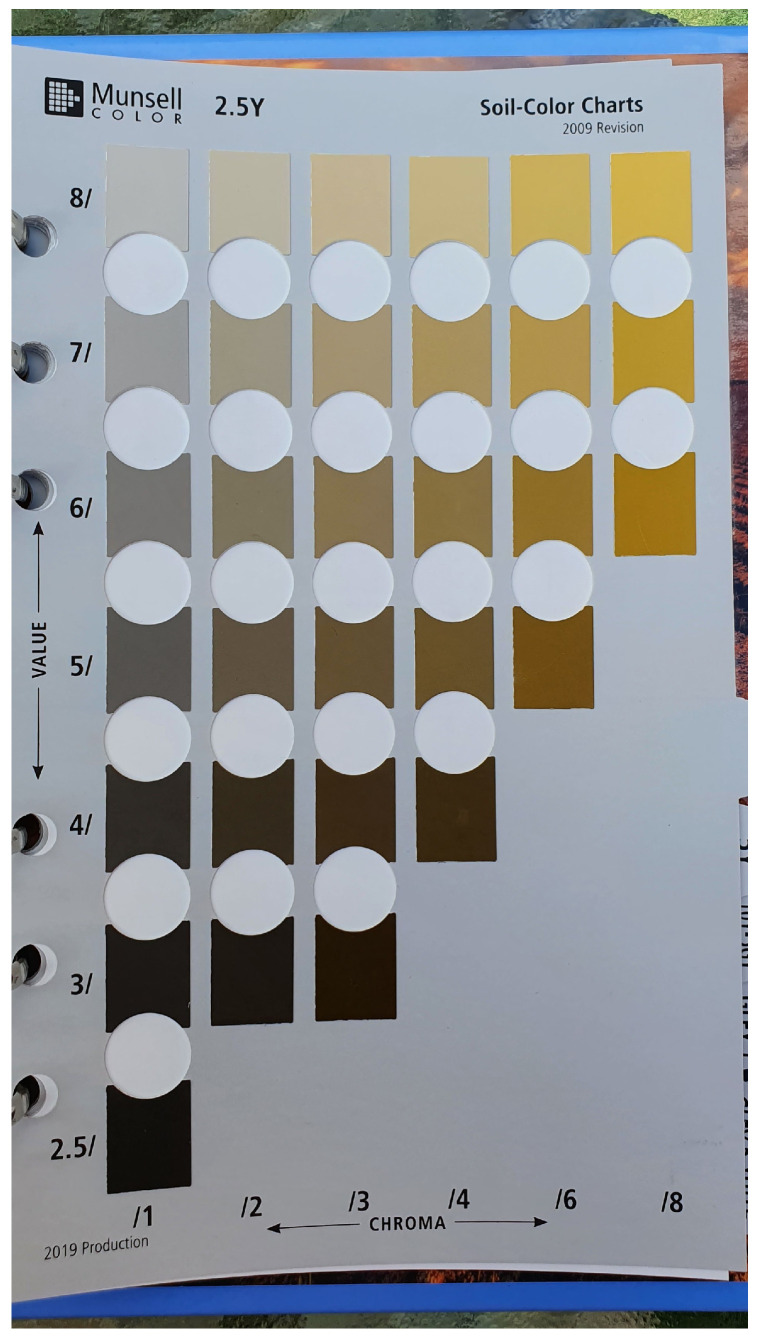
Image of the 2.5Y page of the MSCB, from the data collection process.

Munsell soil colour charts have 13 Hue cards or pages: 5R, 7.5R, 10R, 2.5YR, 5YR, 7.5YR, 10YR, 2.5Y, 5Y, 10Y–5GY, GLEY1, GLLEY2, and WHITE PAGE. Each page contains variations in Value and Chroma in vertical and horizontal directions [10]. Figure 2 is an example page (2.5Y) from the 2009 edition of the MSCB. MSCB is based on the Munsell colour system, and has three properties: Hue, Chroma and Value. Hue represents basic colour, Chroma the intensity, and Value the lightness [47]. Each page of the MSCB has several colour chips, where the page represents Hue, the horizontal numbers are the Value, and the vertical arrays are Chroma. Altogether, the book comprises 443 colour chips, with each representing a unique soil colour.

A Samsung Galaxy S10 smartphone was employed as the data collection tool, as it has a very good camera sensor, produces good quality images [48], and has a 12 MP telephoto lens (45°) [49]. The patching technique was implemented after data preparation, and we compared and analysed both patched data and non-patched data. Following that, K-fold cross-validation was implemented so that the deep learning models proved the best possible accuracy. The accuracy for both page-level and chip-level prediction was established, followed by the top K accuracy as some of the colour chips are spectrally almost identical. Therefore, Top 5 predictions provide much better results with the patching technique.

**Figure 3:**
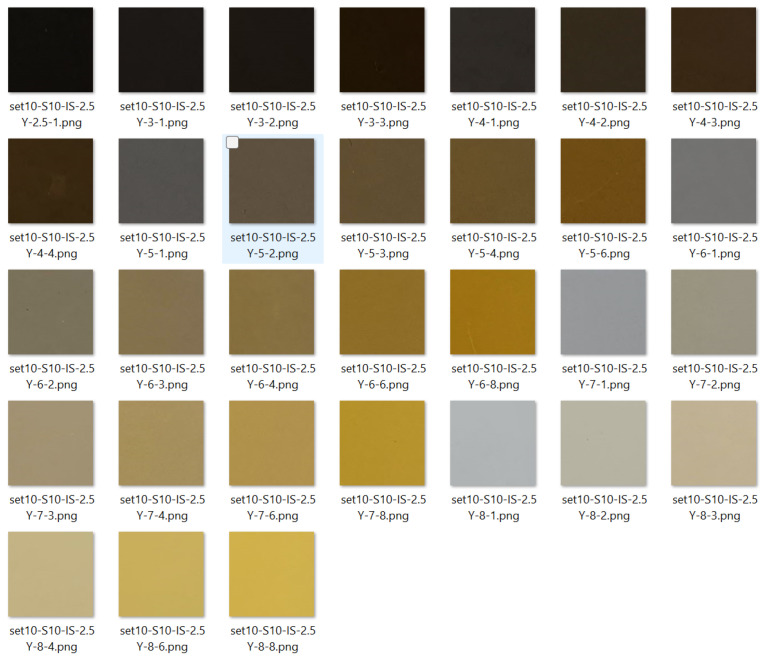
Image of cropped (150 px/150 px) colour chips of the 2.5Y page of the MSCB.

Images of each page were captured at different times of the day to obtain the variation of the sunlight throughout the day using the smartphone. The images were collected every hour from 8 a.m. to 5 p.m. on 20 March 2022. This resulted in 10 images collected for each page of the MSCB. The data collection was performed using an indirect sunlight method, where a shadow was cast on the book to eliminate unnecessary reflection. Captured photos were collected using a free-style approach with some minimal pre-processing before use in deep learning models. The users do not need any external device, camera settings or an outdoor setup to capture the images. The auto camera settings of the smartphone were applied with the smartphone located within 45 to 60 cm distance between the book and the smartphone. This was to mimic likely field conditions where a smartphone user would open the application and take the photo without manually adjusting the camera settings. Each colour chip for MSC prediction was cropped using Photoshop to 150 px/150 px. Figure 2 is an example of the 2.5Y page of the MSCB, and Figure 3 shows the cropped colour chips of the page.

**Figure 4:**
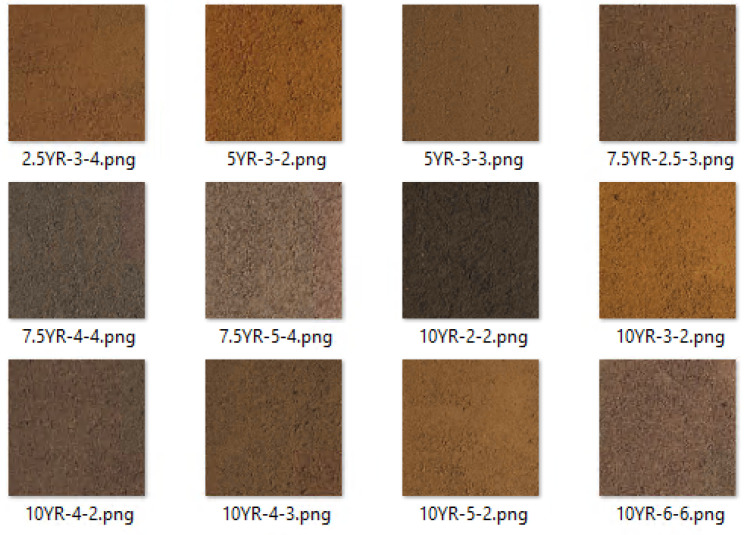
Image of cropped (600 px/600 px) 12 soil samples.

To demonstrate the application of the proposed method in a real-world soil scenario, some soil samples were collected during the National Soil Judging Competition 2023 [50]. Naturally, soils does not have a uniform colour as the MSCB. It contains shadow, texture, dirt, etc. After data collection, the dry soils were then sieved to obtain fine granules to minimize structures and shadows. After that, the same smartphone was used to take pictures of the 12 soils under a constant light condition. Each image was cropped using Photoshop to 600 px/600 px to avoid redundant characters in the images. Figure 4 shows the cropped images of the soil samples. The colours of these 12 samples were determined by a soil scientist, and the colours are: 7.5YR-2.5-3, 5YR-3-3. 2.5YR-3-4, 5YR-3-2, 10YR-4-2, 10YR-4-3, 10YR-5-2, 10YR-3-2, 10YR-2-2, 10YR-6-6, 7.5YR-4-4, and 7.5YR-5-4. Not all of the Hues are prominent in Australia. A study by [51] showed that the 7 most common Hues that are present in Australian top soils are 5Y, 2.5Y, 10YR, 7.5YR, 5YR, 2.5YR, and 10R. For this study, to demonstrate the proposed method, a small number of soil colours were analysed with a constraint experiment to represent real soil. The analysis with real soil will be sturdy when more soil with variation in colour will be collected.

### 2.2. Patching

By using the patching technique, larger images or datasets can be divided into smaller sections or patches. These patches can be used individually in machine learning or deep learning for image classification, segmentation, etc. Patching is often used as a data augmentation technique, as it can effectively increase the amount of data by creating multiple patches for training models. A study by [52] demonstrated that a patch-based vision transformer achieved state-of-the-art performance for image recognition.

**Figure 5:**
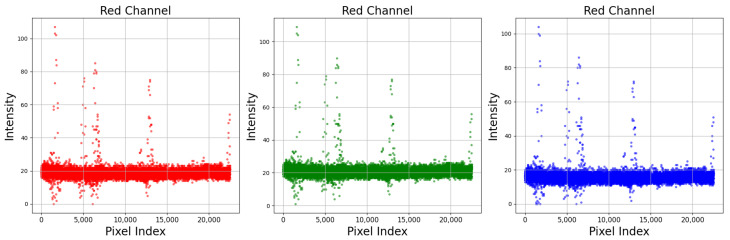
Pixel intensity of GLEY1-2.5-5GY colour chip (150 px/150 px) for RGB colour channel. The intensity of R, G and B channels is not constant in all pixel indexes. The x-axis represents the index of the pixel in the array of pixel values. The y-axis represents the intensity values of the pixel for each respective colour channel (red, green, blue).

**Figure 6:**
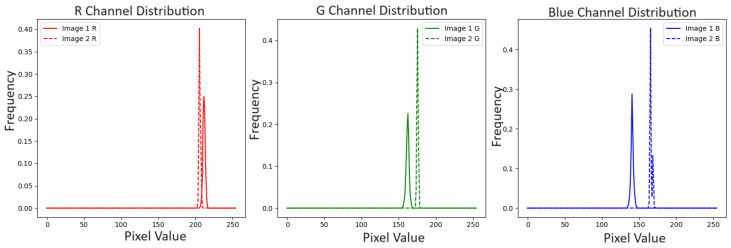
RGB colour difference of two different images(150 px/150 px) of 10R-5-1 colour chip captured at different times. Image1 was captured at 9 a.m. and Image2 captured at 1 p.m. of the same day. The x-axis represents the pixel values (0–255). The y-axis represents the normalized frequency of the pixel values.

The dataset collected for the deep learning models includes 443 classes acquired 10 times to reflect 1 hourly image acquisition and variations in the day. Even then, it is inadequate to train a model properly, as the number of classes is considerably more than the number of samples. Also, the colour chips seem to have a constant colour, but in reality, they have slightly different colour values in all pixels. Figure 5 shows that, for the GLEY1-2.5-5GY colour chip, the values of R, G, and B are not constant. Multiple variations were observed in different pixel indexes. Similarly, Figure 6 shows that images of the same colour chip (10R-5-01), captured at 9 AM and 1 PM are different. Therefore, it can be justified that creating patches of the colour chips will result in many variations of a single image and, thus, this process will enrich the dataset. Also, the deep learning algorithm will learn the patterns of MSC and provide better prediction. The patches can be generated using the following function [53]:

(1) patches=patchify(image_to_patch, (patchheight,patchwidth), step=1)

To eliminate this limitation, a patching method was used, using the Patchify Python library as an augmentation process to generate a large dataset from a smaller dataset. Patchify library splits images into smaller overlapping or non-overlapping patched images [53]. This patching method has two important attributes: patch size (patch height, patch width) and step, shown in Equation (1). As the prepossessed images of colour chips are 150 px/150 px in size, to use Patchify for this study, the desired patch size was set to 100 px/100 px. The step determines how many windows to move after each patch. For this study, the step size was set to 10. The patched images will overlap, as the step size is smaller than the patch size. This method was as follows: because the colours are solid with little variations, this enables us to maximize the datasets. The method delivered 40 patches for each colour chip. The dataset was split into 90% and 10% for calibration and validation purposes. Using the same method, the soil samples (600 px/600 px) were also patched and generated 2601 patches from each image. The soil dataset was then split into 90% and 10% for further training and testing. The reason the soil images produced more patches was that the original soil image was bigger in size than the colour chips from the MSCB.

### 2.3. Applying Deep Learning Techniques for Image Classification

For this study, 4 different deep learning models were trained and tested.

(1) ResNet50 [54]
(2) VGG16 [55]
(3) InceptionV3 [56]
(4) Xception [57]

The models mentioned above are popular deep learning models for image classification using various types of Convolutional Neural Networks (CNNs). All are pre-trained models and have multiple convolutional layers for extracting features from an image. A previous study [58] compared ResNet50 and VGG16 for image classification on 6000 classified images, where ResNet50 performed the best. To compare different CNN architectures, including VGGNET, ResNet, and InceptionV4 (another version of inception v3), the authors of [59] classified soil aggregates obtained from ploughing and produced satisfactory accuracy. Another very useful image classification model is Xception. It has achieved good performance on image classification with transfer learning [60]. Deep learning has also been a popular method in the field of soil classification. Researchers implemented multiple deep learning models, including ResNet50, VGG16, Inception, and Xception, for soil type classification [61].

These 4 deep learning models were selected for this study because of their diverse architectural strengths. All of them are pre-trained models, and have advantages for feature extraction. ResNet50 uses residual connections, and allows the model to learn deeper representations of images, which makes this model suitable for finer soil colour classification [62]. Alternatively, VGG16 uses a simple yet deep architecture. The 16-layer deep model extracts abstract features and improves the ability to differentiate between images. A study [63] demonstrated that classification accuracy improved for image classification using VGG16 as it uses small convolution filters, making it effective for feature extraction. Another well-suited deep learning model for soil colour classification is InceptionV3, because its architecture captures multi-scale features and can distinguish between complex textures and patterns. Researchers of study [64] introduced InceptionV3 and showed its ability to handle multi-scale features and its usefulness for image classification. Lastly, Xcpetion is also effective for soil colour classification due to its depth-wise separable convolution architecture, which allows it to capture finer features with fewer parameters. The researcher of article [65] performed thorough research on this deep learning model and emphasised its ability to capture fine details in images.

This study’s deep learning models were tuned with model setup, callbacks, K-fold cross-validation, metrics and results logging, and final model selection for multi-class image classification.

Model setup:

(1) Base model: first, the base model was set with include_top = False argument, ensuring only the convolutional base was used.
(2) Global average pooling 2D: reduces output to a vector.
(3) Dense layer: connected layer with 1024 units and Relu activation for nonlinearity.
(4) Output layer: final dense layer with softmax activation
(5) Freezing layers: keeping the pre-trained weights intact, it keeps the layers frozen.
(6) Image processing: the image data are pre-processed using ImageDataGnerator.

Callbacks: To monitor the training and improve performance, callbacks were used in the model. Model checkpoints, early stopping, and ReduceLRonPLateau were used to automate the tasks and improve training efficiency and model performance.

K-fold cross validation: this provides a more robust estimation of the model’s performance.

Metrics and result logging: to obtain the results, the test accuracy, Top 5 accuracy, precision, recall, f1-score, and F2-score were calculated to assess the classification models.

### 2.4. K-Fold Cross Validation and Top K Accuracy

The cross-validation technique is used to ensure the effectiveness of a deep learning model. The technique leads to less biased models and ensures maximum dataset observation in the test set [66]. K-Fold Cross validation is a widely used technique for evaluating the performance of a machine learning or deep learning model. The authors of [67] introduced K fold cross-validation to estimate the performance of a statistical learning model. The technique divides datasets into K size folds, and trains on K-1 folds and tests on the remaining dataset, repeating the process K times. The final result is the average metrics from all the folds. This method help mitigate overfitting and provides better model performance by ensuring each data point is used for training and validation.

For this study, the K-Fold cross-validation included 10 splits (K = 10) and employed them in the model to test the model performance. The model is trained and validated in 10 loops by giving the (K-1) folds to train and validate in the Kth fold each time.

For the MSCB, colour chips can be similar and difficult to distinguish using human vision. Therefore, Top 5 accuracy was also calculated, which means the exact colour match can be found in the Top 5 predictions. This study calculated top K accuracy with general accuracy to obtain maximum possibilities, so that the application can provide the archaeologists or farmers with 5 colour options, and they can identify the correct soil colour match from the list of colours.

## 3. Results and Discussion

### 3.1. MSC Prediction on Primary Data

The primary data were first trained and tested with a deep learning technique to analyse the patching method. The primary dataset combined 10 sets of data for the 443 colour chips. Standard deviation, precision, recall, and f1 score are shown for primary and patched data in Table 1, Table 2, Table 3 and Table 4. Test accuracy for both primary and patched data (Table 5 and Table 6) were calculated to assess if the patching method improves accuracy for both page-by-page and chip-by-chip prediction. Standard deviation indicates how scattered the data are in relation to the mean. The value of the standard deviation is expected to be closer to zero. This indicates that the data are closer to the mean, and a higher value means they are further away from the mean [68]. Precision is the measurement of positive prediction of a class for deep learning models, and recall is the true positive rate or the ratio of correctly predicted class among the actual class. Higher precision and recall are desired, as they show that the most positive prediction is correct and has a low false negative rate. A higher f1 score means the deep learning model is predicting a high ratio of positive cases.

#### 3.1.1. Page/Hue Level Prediction

**Figure 7:**
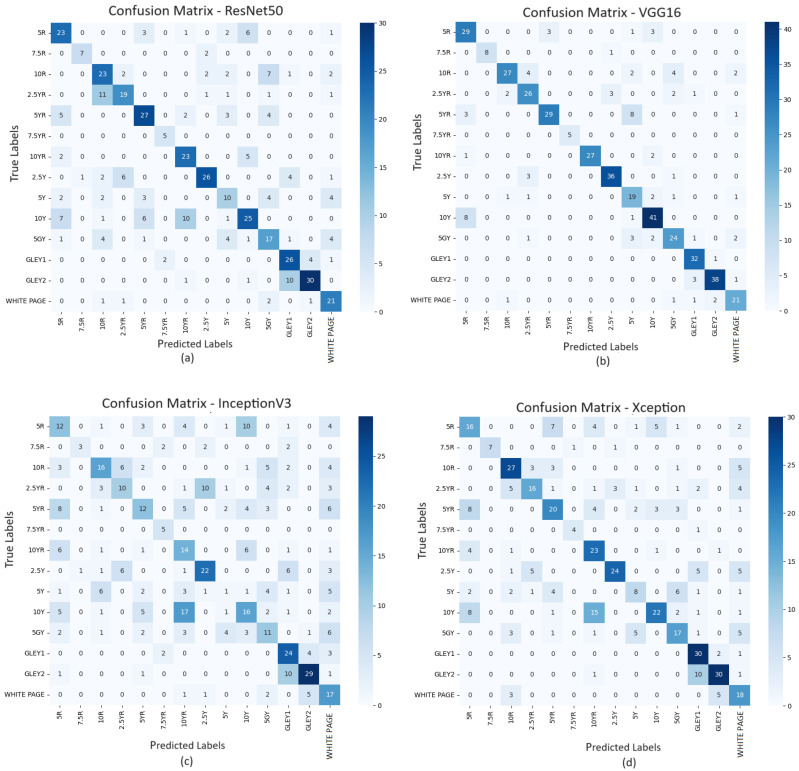
Confusion matrix of page-by-page prediction for ResNet50 (**a**), VGG16 (**b**), InceptionV3 (**c**), and Xception (**d**) with primary dataset.

The first approach of this study is to analyse page-by-page or Hue level of prediction. The dataset used 200 epochs with all four deep learning algorithms. Table 1 suggests standard deviation is high for all four deep learning techniques, data are scattered and many of the predictions are away from the actual values. Also, the precision, recall, and f1 scores are not high. Figure 7 is an example of the confusion matrix of all four techniques. The confusion matrix is a performance measurement for machine learning classification problems where visually the true and false predictions can be shown. It is visible that the trained models could not predict correct Hues/pages with high confidence due to the small dataset available for deep learning to classify an image adequately. Table 5 shows very low accuracy across the deep learning techniques. For this scenario, the ResNet50 performed better than others, and InceptionV3 produced the lowest performance. ResNet50 (65%) and VGG16 (63%) performed better than the two other models for page-level predictions.

#### 3.1.2. Chip-Level Prediction

For chip-level prediction, the dataset was divided into 443 classes, with each class containing 10 images. The approach was follows: Hue level prediction was used to train and test the four deep learning techniques and analyse the results. Table 2 similarly shows that the standard deviation is high, but the precision, recall, and f1 scores are low, and not at all as satisfactory as for page-by-page prediction. The test accuracy is lower than the page-by-page prediction, and none of the deep learning techniques achieved greater than 40% accuracy (Table 5). This is due to the high number of classes and low number of datasets for each class.

### 3.2. MSC Prediction on Patched Data

After obtaining results using the primary dataset, the same above steps were performed with the patched dataset by using the patching method. This method increases the data value by four times that of the primary dataset for both page level and chip level.

#### 3.2.1. Page/Hue-Level Prediction

**Figure 8:**
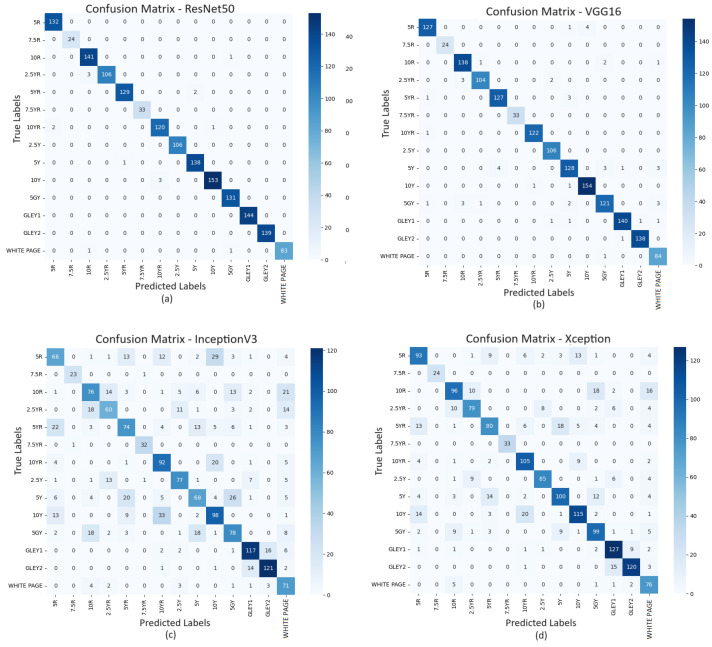
Confusion matrix of page-by-page prediction for ResNet50 (**a**), VGG16 (**b**), InceptionV3 (**c**) and Xception (**d**) with patched dataset.

The page-level prediction for patched data achieved the most favourable results. Table 5 shows that ResNet50 achieved a greater result of 94.53% accuracy, whereas InceptionV3 has an accuracy of 63%. Table 3 shows the standard deviation for ResNet50 is closer to 0, and precision, recall, and f1score are higher than other deep learning techniques. Figure 8 clearly shows that all four techniques managed to perform better with patched data than the primary data (Figure 7) and successful prediction of a higher number of classes.

#### 3.2.2. Chip-Level Prediction

The chip-by-chip prediction for four deep learning techniques with patched data resulted in an accuracy of 95%, and the Top 5 test accuracy is almost 99%, which is expected with ResNet50. Here, ResNet50 also performed much better than the three other deep learning methods. InceptionV3 performed poorly with 56% accuracy, and for the Top 5 accuracy, it achieved 87% accuracy. Both ResNet50 and VGG16 performed similarly, with an accuracy above 90%. Table 4 clearly shows that ResNet50 and VGG16 produced similar and higher precision, recall and f1 scores, and a lower standard deviation.

#### 3.2.3. Prediction on Real Soil Samples

This study determined the MSC from the MSCB, although soil colour determination from real soil is challenging, as the soil has various impurities and is extremely variable for many properties linked to soil colour, such as dirt, texture, structure, etc. For this study, 12 soil samples were collected from a soil pit in Darwin as representations of real soil. Figure 4 shows that some colours are very close to each other. This is very common because the Munsell soil colour shows that the colour chips are very close to each other, and sometimes it is very hard to differentiate between two colours. Even two soil scientists may determine the colours differently, as it entirely depends on human eyes and is hard to differentiate.

The vast diversity of soil types and colours globally poses a significant challenge, as the number of possible variations is immense. Collecting soil samples globally and performing colour characterization is a resource-intensive task requiring significant time and collaboration. To demonstrate the proposed method, a limited set of soil colours was analysed through a controlled experiment to reflect real-world soil conditions. The analysis is expected to become more robust as additional soil samples with diverse colour variations are collected.

The same chip-by-chip prediction method, incorporating the patching algorithm, was applied to demonstrate the proposed method’s potential for real-world soil prediction. Soil samples, shown in Figure 4, were randomly cropped to create a soil testing dataset, which was used to evaluate the proposed method across four deep learning techniques. The models developed in the chip-level prediction phase (Section 3.1.2) were tested on this real soil dataset. The deep learning models were trained exclusively using MSCB colour chips and tested on real soil samples to assess their effectiveness in real-world soil. As shown in Table 7, ResNet50 outperformed the other techniques, achieving 63% accuracy on Top 5 predictions, followed by VGG16 with 53% accuracy. Inception V3 and Xception demonstrated similar performances on the real soil samples, with 48% accuracy. This accuracy is expected, as real soil samples exhibit a wide range of characteristics, while the MSCB colour chips consist of uniform solid colour chips.

The proposed method with the combination of deep learning and patching can determine MSC both page-by-page and chip-by-chip. At this stage, the method (deep learning and patching) was evaluated by comparing it with the primary dataset. Furthermore, smartphone-captured images were used, which can be easily captured by users for soil colour determination. The proposed approach with the combination of deep learning and patching methods shows promising results. The patching method is a way to generate many images from a single image, and also improves the results for soil colour prediction. For that reason, page-by-page prediction is higher than the chip-by-chip prediction as pages contain original data as well as patched data.

In future studies, the aim is to determine soil colour from soil samples using the proposed approach of this research with more soil samples. The key findings are summarized as follows:

- According to the analysis and data, between all four deep learning algorithms, ResNet50 outperformed all other models across all combinations of page-by-page and chip-by-chip with primary data and patched data. Inception V3 performed lower than others.
- ResNet50 and VGG16 performed similarly when the dataset is bigger.
- The patching method as a data augmentation technique for deep learning achieved significant results for MSC prediction. The accuracy increases from 65% to 95% for page-level prediction and from 40% to 95% for chip-level prediction.
- The most accurate prediction was achieved when the Top 5 predictions were calculated. With patching and ResNet50, the proposed method achieved a 99% prediction rate.
- The proposed method is effective on real soil samples, and more soil samples with variations in colours will achieve better prediction.

## 4. Conclusions

This study evaluated different deep learning methods to predict MSC using a data preparation technique. As anticipated, the performance of deep learning models was dependent on large data collections. Patching as an image augmentation method performed well with all four deep learning techniques. Among all of the deep learning techniques, ResNet50 performed the best. For both page-by-page and chip-by-chip, the accuracy is 95%, and the Top 5 test accuracy is 99%. VGG16 also performed similarly to the patching method, and showed 98% accuracy for the Top 5 predictions at the chip level. The ultimate goal of this study was to predict the MSCB colour chip with the patching method using deep learning. This study proves that the patching method combined with ResNet50 provides the most effective prediction for Munsell soil colour from MSCB. However, the dataset was collected on a single day for this study; the trained deep learning model can improve with more data collected on different days and weather conditions. Also, for this study, only one smartphone (Samsung Galaxy S10) with auto-camera settings was used. Different camera sensors produce slightly different images, and the current market has various kinds of smartphones. Furthermore, the proposed method was evaluated on a limited set of soil samples to illustrate its applicability in practical soil scenarios, suggesting its effectiveness as more soil data becomes available. Moreover, to analyse soil colour from actual soil, comprehensive and diverse soil samples need to be collected by following proper protocol, imaging techniques and ethical approval. Furthermore, soil as a medium does not frequently have solid colours like Munsell soil colour chips; rather, it can be combinations of colours and other confounding properties, such as texture and sample presentation, which require further examination with different smartphones under various environmental conditions.

## Figures and Tables

**Table 1:** Test accuracy of primary dataset—page-by-page.

Primary—Page-by-Page				
Model	Standard Deviation	Precision	Recall	f1_Score
ResNet50	3.21	0.65	0.65	0.65
VGG16	1.84	0.63	0.63	0.62
inceptionV3	3.59	0.44	0.44	0.42
Xception	3.02	0.51	0.50	0.49

**Table 2:** Test accuracy of primary dataset—chip-by-chip.

Primary—Chip-by-Chip				
Model	Standard Deviation	Precision	Recall	f1_Score
ResNet50	2.47	0.44	0.39	0.39
VGG16	2.54	0.42	0.38	0.37
inceptionV3	1.79	0.67	0.22	0.21
Xception	2.3	0.21	0.20	0.19

**Table 3:** Test accuracy of patched dataset—page-by-page.

Patched—Page-by-Page				
Model	Standard Deviation	Precision	Recall	f1_Score
ResNet50	0.96	0.95	0.95	0.95
VGG16	1.13	0.91	0.90	0.90
InceptionV3	1.72	0.63	0.63	0.63
Xception	1.92	0.70	0.70	0.70

**Table 4:** Test Accuracy of patched dataset—chip-by-chip.

Patched—Chip-by-Chip				
Model	Standard Deviation	Precision	Recall	f1_Score
ResNet50	0.69	0.97	0.95	0.95
VGG16	0.94	0.95	0.93	0.93
InceptionV3	1.91	0.63	0.56	0.56
Xception	1.4	0.70	0.66	0.65

**Table 5:** Test accuracy comparison of 4 deep learning techniques on primary and patched datasets.

Model	Primary Page-by-Page Test Accuracy	Primary Chip-by-Chip Test Accuracy	Patched Page-by-Page Test Accuracy	Patched Chip by Chip Test Accuracy
ResNet50	**64.81%**	**39.48%**	**94.53%**	**95.23%**
VGG16	62.55%	37.95%	90.44%	92.82%
InceptionV3	43.88%	21.81%	63.02%	56.38%
Xception	49.59%	19.53%	69.73%	65.73%

**Table 6:** Top 5 Test accuracy comparison of 4 deep learning techniques on primary and patched datasets.

Model	Primary Page-by-Page Top 5 Test Accuracy	Primary Chip-by-Chip Top 5 Test Accuracy	Patched Page-by-Page Top 5 Test Accuracy	Patched Chip-by-Chip Top 5 Test Accuracy
ResNet50	**98.19%**	**73.81%**	**99.87%**	**98.73%**
VGG16	96.25%	70.09%	99.68%	98.34%
InceptionV3	90.43%	55.62%	96.88%	87.37%
Xception	93.34%	49.62%	98.10%	90.07%

**Table 7:** Test accuracy comparison of 4 deep learning techniques trained on the patched chip-by-chip MSCB dataset and tested on the real soil dataset.

Model	Average Test Accuracy	Average Top 5 Test Accuracy
ResNet50	19.58%	62.81%
VGG16	8.65%	53.02%
Inception V3	9.06%	47.81%
Xception	11.67%	48.02%

## Data Availability

Data are contained within the article.
